# SHP1-mediated cell cycle redistribution inhibits radiosensitivity of non-small cell lung cancer

**DOI:** 10.1186/1748-717X-8-178

**Published:** 2013-07-10

**Authors:** Rubo Cao, Qian Ding, Pindong Li, Jun Xue, Zhenwei Zou, Jing Huang, Gang Peng

**Affiliations:** 1 Cancer Center of Union Hospital, Tongji Medical College, Huazhong University of Science and Technology, No. 1227 Jiefang Dadao, Wuhan 430022, China

**Keywords:** Non-small cell lung cancer, SHP1, Radiosensitivity, Cell cycle

## Abstract

**Background:**

Radioresistance is the common cause for radiotherapy failure in non-small cell lung cancer (NSCLC), and the degree of radiosensitivity of tumor cells is different during different cell cycle phases. The objective of the present study was to investigate the effects of cell cycle redistribution in the establishment of radioresistance in NSCLC, as well as the signaling pathway of SH2 containing Tyrosine Phosphatase (SHP1).

**Methods:**

A NSCLC subtype cell line, radioresistant A549 (A549S1), was induced by high-dose hypofractionated ionizing radiations. Radiosensitivity-related parameters, cell cycle distribution and expression of cell cycle-related proteins and SHP1 were investigated. siRNA was designed to down-regulate SHP1expression.

**Results:**

Compared with native A549 cells, the proportion of cells in the S phase was increased, and cells in the G0/G1 phase were consequently decreased, however, the proportion of cells in the G2/M phase did not change in A549S1 cells. Moreover, the expression of SHP1, CDK4 and CylinD1 were significantly increased, while p16 was significantly down-regulated in A549S1 cells compared with native A549 cells. Furthermore, inhibition of SHP1 by siRNA increased the radiosensitivity of A549S1 cells, induced a G0/G1 phase arrest, down-regulated CDK4 and CylinD1expressions, and up-regulated p16 expression.

**Conclusions:**

SHP1 decreases the radiosensitivity of NSCLC cells through affecting cell cycle distribution. This finding could unravel the molecular mechanism involved in NSCLC radioresistance.

## Background

Lung cancer is one of the malignant tumors with the fastest-growing morbidity and mortality in China. Non-small cell lung cancer (NSCLC) accounts for 80-85% of all lung cancer cases, and has a 5-year survival rate of less than 15% [1]. Radiations therapy has been regarded as the main treatment strategy for NSCLC for a long time. However, radioresistance is the key issue limiting the effects of radiations [2]. Due to the presence of tumor cells heterogeneity, malignant cells might exhibit different degrees of radiosensitivity even when they are from the same histological differentiation status. Radioresistant cells can survive to radiotherapy, which in turn induces the local recurrence of NSCLC [3,4]. Many recent advances in functional imaging and radiations therapy technology, such as intensity-modulated radiation therapy (IMRT) and image-guided radiation therapy (IGRT), allowed for improved treatments. However, strategies for overcoming the radioresistance-related treatment failure in NSCLC are still largely unknown [5].

It has been found that the intrinsic radiosensitivity of cells subpopulations present in low- and high-radiosensitive subsets is different. This difference is based on the level of hypoxia, DNA repair capacity, the number of dividing and apoptotic cells and cell cycle phases. Among these, the regulation of cell cycle might play a major role in this process [6,7].

The biological behavior of NSCLC is closely related to a variety of cellular signal transduction pathways [8-12]. Protein tyrosine kinase (PTK) and protein tyrosine phosphatase (PTP) are two important signals mediating tyrosine phosphorylation and dephosphorylation, respectively. PTK, PTP and their substrates act for signal transduction. Previous studies have shown [13,14] that multiple tyrosine phosphorylation proteins play a pivotal role during the development of diseases. Indeed, the protein tyrosine phosphatase SHP1 is a key regulator that mediates the level of intracellular phosphorylation. The gene encoding this protein is 17 kb long and contains 17 exons. The interaction of ligand and its receptor on the cell membrane can induce the receptor dimerization after cytokines stimulation. The receptor and its coupled JAK kinases can then be activated via tyrosine phosphorylation. Meanwhile, the activated SH2 domain of SHP1 is able to catalyze JAKs or to induce tyrosine dephosphorylation of other tyrosine kinases (such as Src and c-fms). This induces a stop or a decrease in the kinase activity, negatively regulates cellular signal transduction, and inhibits cell proliferation [6,7,15-23]. Recent studies showed that SHP1 regulates cell cycle, proliferation and tumor progression by modulating cell cycle machinery through cyclin-dependent kinase 2 (CDK2), p27 and CyclinD1 [17]. In addition, the inhibition of SHP1 in prostate cancer cells have been shown to induce G0/G1 phase cell cycle arrest and to change some cell cycle machinery, such as down-regulation of p27, CDK2 and CDK6 [18]. Taken together, SHP1 is well-known to be associated with cell cycle regulation.

We hypothesized that SHP1 might affect the radiosensitivity of NSCLC by modulating cell cycle. Thus, SHP1 might serve as a potential target for regulating the radioresistance of NSCLC. In this study, we first established an A549 radioresistant subtype cell line (A549S1). We further demonstrated the phenomenon of G0/G1 and S phase arrest in this cell line, which was demonstrated by the data showing an increase and a decrease in the proportion of cells in the S and G0/G1 phase, respectively. Meanwhile, we demonstrated that the cellular levels of SHP1, CDK4 and CylinD1 in this cell line were increased, while the level of p16 was significantly decreased. Finally, the inhibition of SHP1 expression in A549S1 cells up-regulated their radiosensitivity and induced G0/G1 phase arrest. Taken together, our results provide the molecular basis for NSCLC radioresistance that can be leveraged in order to unravel the theoretical basis for improving the radiotherapy effectiveness in NSCLC.

## Materials and methods

### Reagents

The RPMI-1640 and G418 culture medium were purchased from Gibco (GIBCO, Invitrogen Inc., Carlsbad, CA, USA). Fetal bovine serum (FBS) was purchased from Hangzhou Sijiqing Biological Engineering Materials Co., Ltd. (Hangzhou, China). Trypsin, propidium (PI) and RNA enzyme were from Sigma (St. Louis, MO, USA). Lipofectamine 2000, Trizol, OPTI-MEM I and MMLV reverse transcriptase were from Invitrogen (Carlsbad, CA, USA). Taq DNA polymerase and Oligo dT primers were from Invitrogen (Carlsbad, CA, USA). dNTPs and DNA/protein molecular weight standards were purchased from Fermentas Inc. (Thermo Fisher Scientific, Waltham, MA, USA). Protein lysis buffer and BCA protein assay kit were from the Beyotime Institute of Biotechnology (Wuhan, China). Protease inhibitors were from Roche (Basel, Switzerland). Rabbit anti-human SHP-1, SHP-2, p16, CDK4 and Cylin D1 monoclonal antibodies were purchased from Cell Signaling Technology (Danvers, MA, USA). The rabbit anti-human GAPDH antibody was from Santa Cruz Biotechnologies Inc. (Santa Cruz, CA, USA). HRP-conjugated goat anti-rabbit secondary antibody IgG was purchased from Beijing Zhongshan Golden Bridge Co., Ltd. (Beijing, China). The ECL chemiluminescence reagent was from Pierce Chemicals (Rockford, IL, USA).

### Cell culture

The human NSCLC cell line A549 was purchased from the American Type Culture Collection (ATCC). Cells were cultured in RPMI1640 medium containing 10% FBS, 100 IU penicillin and 100 μg/mL streptomycin and incubated at 37°C and in a 5% CO_2_ humidified incubator.

### Establishment of the NSCLC radioresistant subtype cell line

The A549S1 NSCLC radioresistant subtype cell line was established as previously reported [24]. Briefly, 6 MV ionizing radiations from a Siemens Primus H high-energy linear accelerator (Siemens, Erlangen, Germany) was used for irradiation of A549 cells at a field of 10 × 10 cm and a SSD of 100 cm, with an absorption dosage of 200 cGy/min. A thick 1.5-cm block was used to cover culture bottles for compensation. Parental A549 cells in the logarithmic growth phase were randomly divided into two groups (n = 3 per group). Each group received an irradiation dose of 6 or 2 Gy/fraction, which was repeated for five or fifteen fractions, respectively. After each fraction, the cells were passaged at the end of the logarithmic growth phase. Two cell clones were obtained from the surviving cells, which were named A549S1 and A549S2, and were used to investigate the radiosensitivity for three months without irradiation.

### The construction of PGCsiRNA

The SHP-1 transcript (NM_080549.3) was found on the NCBI website and siRNA sequences were designed according to the general international online design software guidelines (http://www.ambion.com/techlib/misc/siRNAfinder.html). siRNA sequences are listed in Table 1. pGCsiRNA-NC was used as the negative control with 16 consecutive bases that did not have any homology with the target gene. pGCsiRNA1907, pGCsiRNA774 and pGCsiRNA-NC (GenePharma, Shanghai, China) were transiently transfected into the parental A549 cells using lipofectamine 2000. The interference efficiency was examined by RT-PCR and Western blot 48 h after transfection.

**Table 1:** **siRNA sequences for *SHP1 *gene**

**Plasmids**	**siRNA sequences (5’-STEM-Loop-STEM-3′)**	**Transcriptional start site**
pGCsiRNA1907	5′-TCCCGACAACACAATACCAGATAAATTC	1907
	AAGAGATTTATCTGGTATTGTGTTGTCTTT-3′	
pGCsiRNA774	5′-TCCCGTCCCATTACTACTGTTCCAATTC	774
	AAGAGATTGGAACAGTAGTAATGGGACTT-3′	
pGCsiRNA-NC	5′-TCCCTTCTCCGAACGTGTCACGTTTC	
	AGAGAACGTGACACGTTCGGAGAATT-3′	

### Measurement of mRNA transcription by Real-time RT-PCR

Total RNA was extracted from the pGCsiRNA1907, pGCsiRNA774 and pGCsiRNA-NC cell groups using Trizol, according to the manufacturer’s instructions. RNA purity was assessed by A260/A280. Total RNA (1 μg) was reverse transcribed into cDNA using primer Oligo dT and MMLV reverse transcriptase (Promega, CA, USA). Specific mRNA quantification was performed by real-time PCR using SYBR® Green master mixes (Life Technologies, NY, USA) in ABI PRISM® 7900HT Sequence Detection System (Life Technologies), according to the manufacturer’s instructions. SHP-1 and glyceraldehyde-3-phosphate dehydrogenase (GAPDH) primers were designed using the Primer Premier 5.0 software. The gene-specific primers used were: SHP-1 forward: 5′-AGA AGC AGG AGT CCG AGG AT-3′, reverse: 5′- GCT GTG GTC AAA GGG GAG AA -3′; GADPH forward: 5′- CTC CTC CTG TTC GAC AGT CAG C -3′, reverse: 5′- CCC AAT ACG ACC AAA TCC GTT -3′. PCR reactions involved 45 cycles of 95°C for 30 s, 60°C for 60 s. The Ct value was defined as the number of PCR cycles in which the fluorescence signal exceeded the detection threshold value. First, ΔCt = Ct Gene - Ct GAPDH. Then, ΔΔCt = ΔCt treated - ΔCt control. Lastly, 2^-ΔΔCt^ was calculated to represent the relative mRNA expression of target genes. GAPDH was used as internal control.

### Western blot

Cell lysates were prepared using the protein lysis buffer, according to the manufacturer’s instructions. Following extraction, protein concentration was determined by a BCA assay. A total of 25 μg protein from each sample was loaded into a 10% SDS-PAGE, followed by a transfer to PVDF membrane (Millipore Corporation, Billerica, MA). Membranes were blocked with 5% non-fat dry milk overnight. They were then incubated with rabbit anti-human SHP1, SHP2, p16, CDK4, GAPDH and CylinD1 antibodies at 4°C overnight. After washing, membranes were incubated with horseradish peroxidase-conjugated goat anti- rabbit IgG for 1 h. The ECL chromogenic agent was used to develop the membranes and the optical density of the bands was analyzed using the Image J software (National Institutes of Health, Bethesda, MD, USA).

### Construction and identification of A549 cells stably transfected plasmids

One day before transfection, A549S1 cells were trypsin-digested and counted. Cells were plated in 6-well plates, and the transfection was conducted when they reached an 80-90% confluence. Cells were plated in 2 ml of DMEM medium containing serum without antibiotics. A total of 2 μg pGCsiRNA1907 was diluted in 250 μl of OPTI-MEM I medium, mixed gently and left at room temperature for 5 min. In the meantime, 5 μl of Lipofectamine 2000 was diluted in 250 μl of OPTI-MEM I medium, mixed gently and left at room temperature for 5 min. The diluted pGCsiRNA1907 and Lipofectamine2000 (250 μl of each) were gently mixed and left at room temperature for 20 min. The mixture was then transferred to the cell culture plates, and mixed gently. After 24 h of culture in a CO_2_ incubator at 37°C, cells were diluted, passaged into three culture plates (10 cm diameter) and treated with the G418 antibiotic (500 μg/ml) after 24 h. Empty vector and blank controls without plasmid DNA were used as negative controls.

Following two weeks of culture in DMEM medium containing G418 (500 μg/ml) and 10% FBS, the formation of monoclonal cell masses tranfected with target genes or empty vector was observed. However, all cells without plasmid DNA transfection died. Monoclonal cell masses were digested with trypsin and transferred into 6-well plates (2 ml DMEM mediun containing 500 μg/ml of G418 and 10% of FBS were added to each plate in advance). Between 12 and 24 clone cell masses were picked up from each transfection system. Cells were passaged into a T25 flask upon reaching an 80-90% confluence and collected upon reaching a 100% confluence. Half of the cells were used to extract RNA for RT-PCR analysis of the target gene expression and the other half was used for cryopreservation. Cells with expression of the target gene were cultured for three months using a compressing model and were stably transfected with the plasmid or empty vector; they were named A549S1/siSHP1 and A549S1/siMock, respectively.

### Clone formation assay for cell survival fraction analysis

A single cell suspension was prepared from the cells in their logarithmic growth phase using 0.25% trypsin for digestion. Cells were seeded in 6-well plates and received a single-dose irradiation of 0, 2, 4, 6, 8 and 10 Gy after a 12 h culture adherence. Two weeks later, cells were treated with 4% paraformaldehyde and were stained using Giemsa. Cell survival fraction (SF) was calculated based on the following formula: SF = colony numbers in experimental group/(Plating number × Plating efficiency (PE)). PE = (colony numbers in control group/Plating efficiency (PE)) × 100%. Cell survival curve was obtained by the single-hit multi-target model (S = l-(1-e-D0/D)N) using Sigma Plot 2001 Demo version. The parameters of cell survival fraction, mean lethal dose value (D0), quasithreshold dose (Dq) and extrapolation number (N) were calculated with a 2 cGy irradiation dose (SF2). Higher values of SF2, D0, Dq and N indicated a higher radiosensitivity.

### Cell cycle analysis by flow cytometry

A single cell suspension was made from cells in their logarithmic growth phase using 0.25% trypsin for digestion. Cells were placed in precooled 70% ethanol at −20°C for fixation overnight. Cells were then washed in PBS and digested using RNA enzyme. PI was added into the cells at a final concentration of approximately 60 μg/ml. Cells were incubated in the dark, and the cell cycle phases were examined by flow cytometry (Beckman, USA). All experiments were conducted in triplicate.

### Statistical analysis

Statistical analysis was performed using SPSS 17.0 (SPSS Inc., Chicago, IL, USA). Significant differences between the groups were determined by the Student’s *t*-test. A P-value <0.05 was considered to be statistically significant (*P* < 0.05).

## Results

### Radiosensitivity changes in A549S1 and A549S2 cells

**Figure 1:**
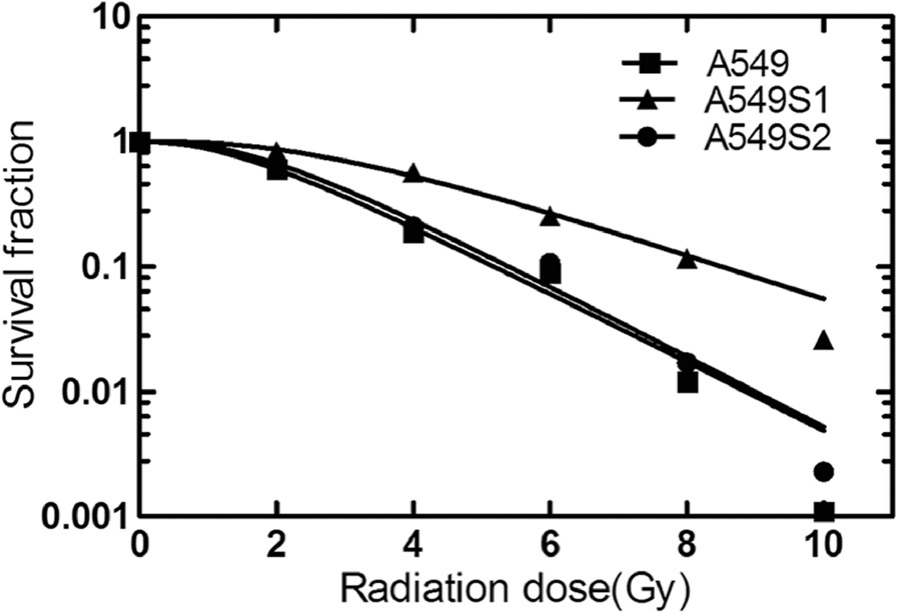
**Survival curves of A549, A549S1 and A549S2 cells.** Cell survival curves were obtained using the single-hit multi-target model (S = l-(1-e-D0/D)N) and the parameters of cell survival fraction in 2Gy irradiation dose (SF2), mean lethal dose value (D0), quasithreshold dose (Dq) and extrapolation number (N) were calculated.

Clone formation assay was used to examine the survival fraction of A549, A549S1 and A549S2 cells following treatment with ionizing radiations. Results showed that irradiation induced cell death in an exponential manner (Figure 1). Table 2 shows the cellular radiosensitivity parameters. SF2, D0, Dq and N were increased in A549S1 cells. Moreover, the plateau phase of the cell survival curve in A549S1 cells was also enhanced, suggesting a higher radioresistance in A549S1 cells compared with A549 cells. However, there were no significant differences between A549S2 and A549 cells in terms of radiosensitivity parameters and of the survival curves. These results suggest that high-dose hypofractionated irradiation could induce the formation of radioresistant NSCLC cells, displaying a higher radioresistance.

**Table 2:** Radiosensitivity parameters of different cell lines

**Radiosensitivity parameters**	**A549**	**A549S1**	**A549S2**
SF2	0.60	0.83	0.67
D_0_	1.76	2.01	1.65
D_q_	1.54	1.98	1.44
N	2.9	4.31	2.38

SF2: cell survival fraction in 2Gy irradiation dose; D0: mean lethal dose value; Dq: quasithreshold dose; N: extrapolation number (N).

### Radiosensitivity of A549 and A549S1 cell lines following a three-month culture

**Figure 2:**
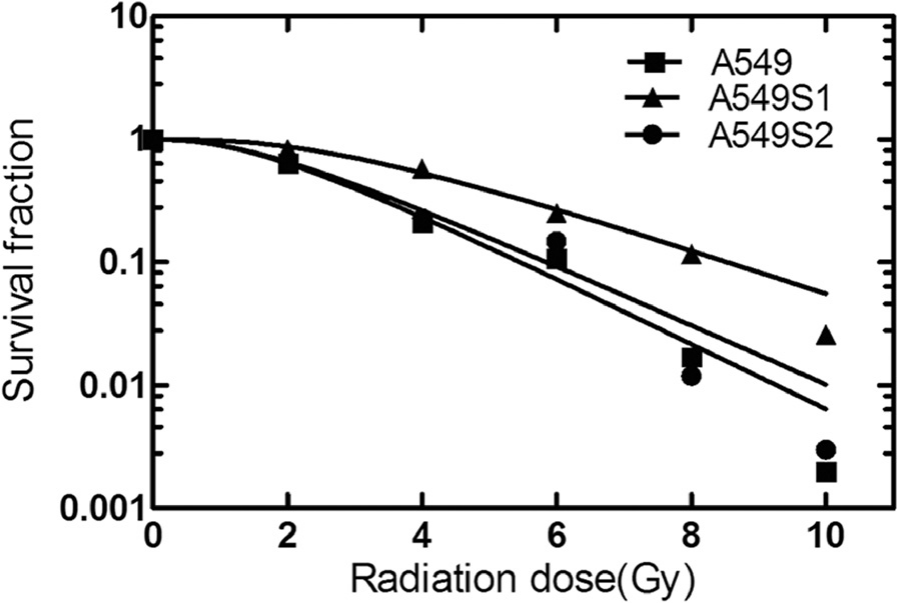
**Survival curves of A549, A549S1 and A549S2 cells after three months of culture.** Cell survival curves were obtained using the single-hit multi-target model (S = l-(1-e-D0/D)N) and the parameters of SF2, D0, Dq and N were calculated.

After radioresistance establishment, A549S1 and A549S2 cells were cultured for three months, and survival fraction was determined by the clone formation assay followed by obtaining cell survival curve as shown in Figure 2. SF2, D0, Dq and N radiosensitivity parameters are shown in Table 3. Results showed that compared with A549 cells, A549S1 cells maintained their radioresistant property longer, even after a three-month culture.

**Table 3:** Radiosensitivity parameters of different cell lines after three months of culture

**Radiosensitivity parameters**	**A549**	**A549S1**	**A549S2**
SF2	0.64	0.83	0.66
D_0_	1.45	2.11	1.43
D_q_	1.55	2.37	1.65
N	2.31	4.32	2.43

SF2: cell survival fraction in 2Gy irradiation dose; D0: mean lethal dose value; Dq: quasithreshold dose; N: extrapolation number (N).

### Cell cycle changes in A549, A549S1 and A549S2 cell lines

**Figure 3:**
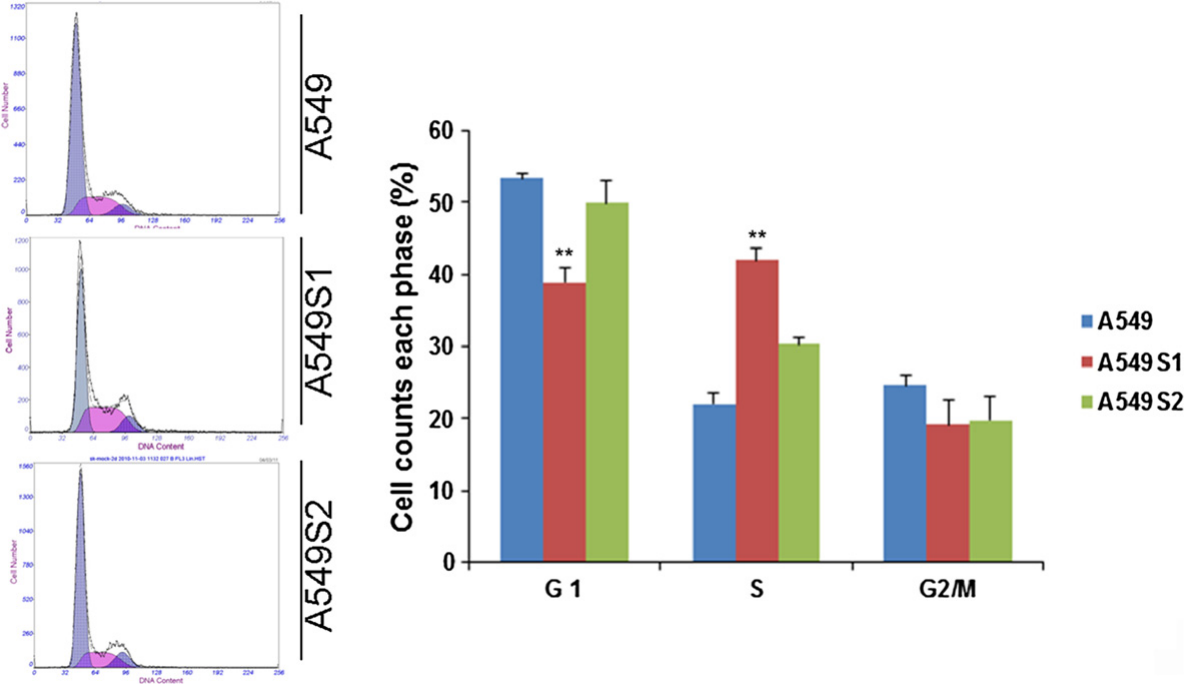
**Assessment of cell cycle in A549, A549S1 and A549S2 cells.** Following culture for three months, cell cycle progression of A549, A549S1 and A549S2 cells were detected by FACS analysis (**P < 0.01 vs. A549 cells).

Cell cycles of A549S1 and A549S2 following a three-month culture are shown in Figure 3. The proportions of A549 cells in G0/G1, S and G2/M phase were 53.4 ± 0.8%, 22.0 ± 1.5% and 24.6 ± 1.5%, respectively. These proportions were changed in A549S1 cells to 38.9 ± 1.9%, 42.0 ± 1.7% and 19.1 ± 3.5%, respectively. When compared to A549 cells, the proportion of A549S1 cells in G0/G1 phase were significantly decreased, while significantly increased for cells in S phase (P <0.01). The proportion of A549S2 cells in G0/G1, S and G2/M phase were 50.0 ± 3.1%, 30.4 ± 0.8% and 19.8 ± 3.3%, respectively. There was no significant differences when these proportions were compared to those of A549 cells (P > 0.05).

### SHP1, SHP2, p16, CDK4 and Cyclin D1 protein levels changes in A549, A549S1 and A549S2 cells

**Figure 4:**
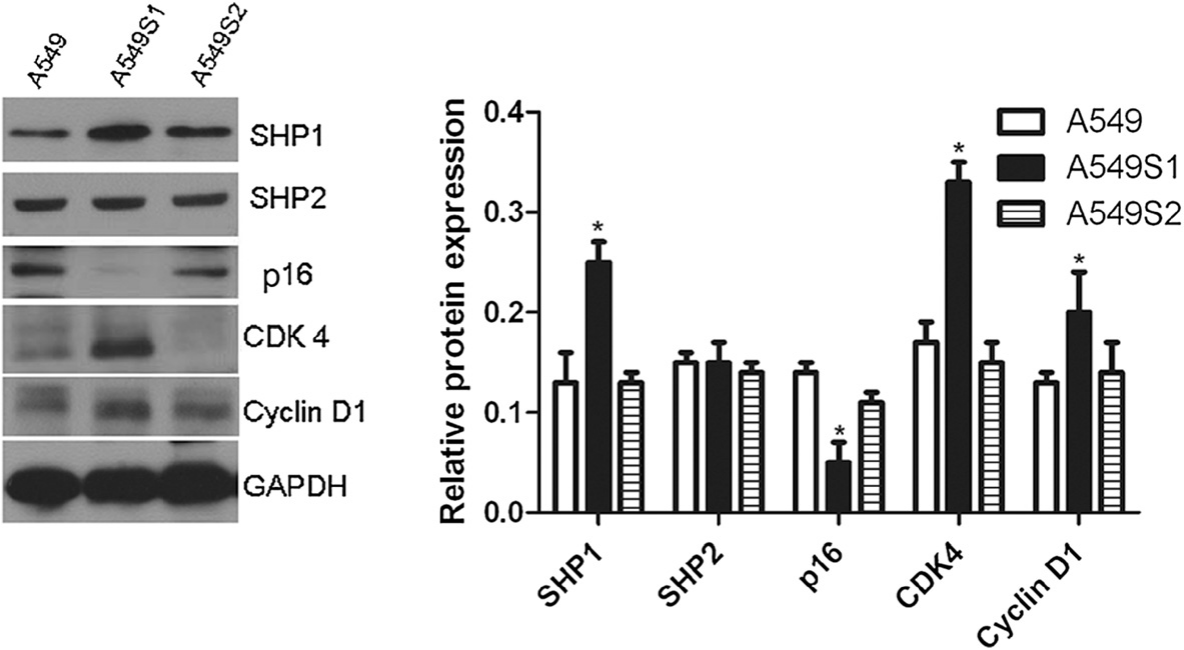
**Protein level changes in SHP1, SHP2, p16, CDK4 and Cyclin D1 in radioresistant cells.** A549S1 and A549S2 cells were cultured for three months. Cellular protein levels of SHP1, SHP2, p16, CDK4 and Cyclin D1 were examined by western blot. GAPDH was used as an internal control (*P < 0.05; **P < 0.01 vs. A549 cells).

As shown in Figure 4, expression levels of SHP1, CDK4 and CylinD1 were significantly increased, while p16 was decreased in A549S1 cell when compared with A549 cell (P < 0.05). There was no significant difference in SHP2 expression between A549S1 and A549 cells (P > 0.05).

### Efficiency of SHP1 siRNA in pGCsiRNA774 and pGCsiRNA1907 plasmids in A549S1 cells at mRNA and protein levels

**Figure 5:**
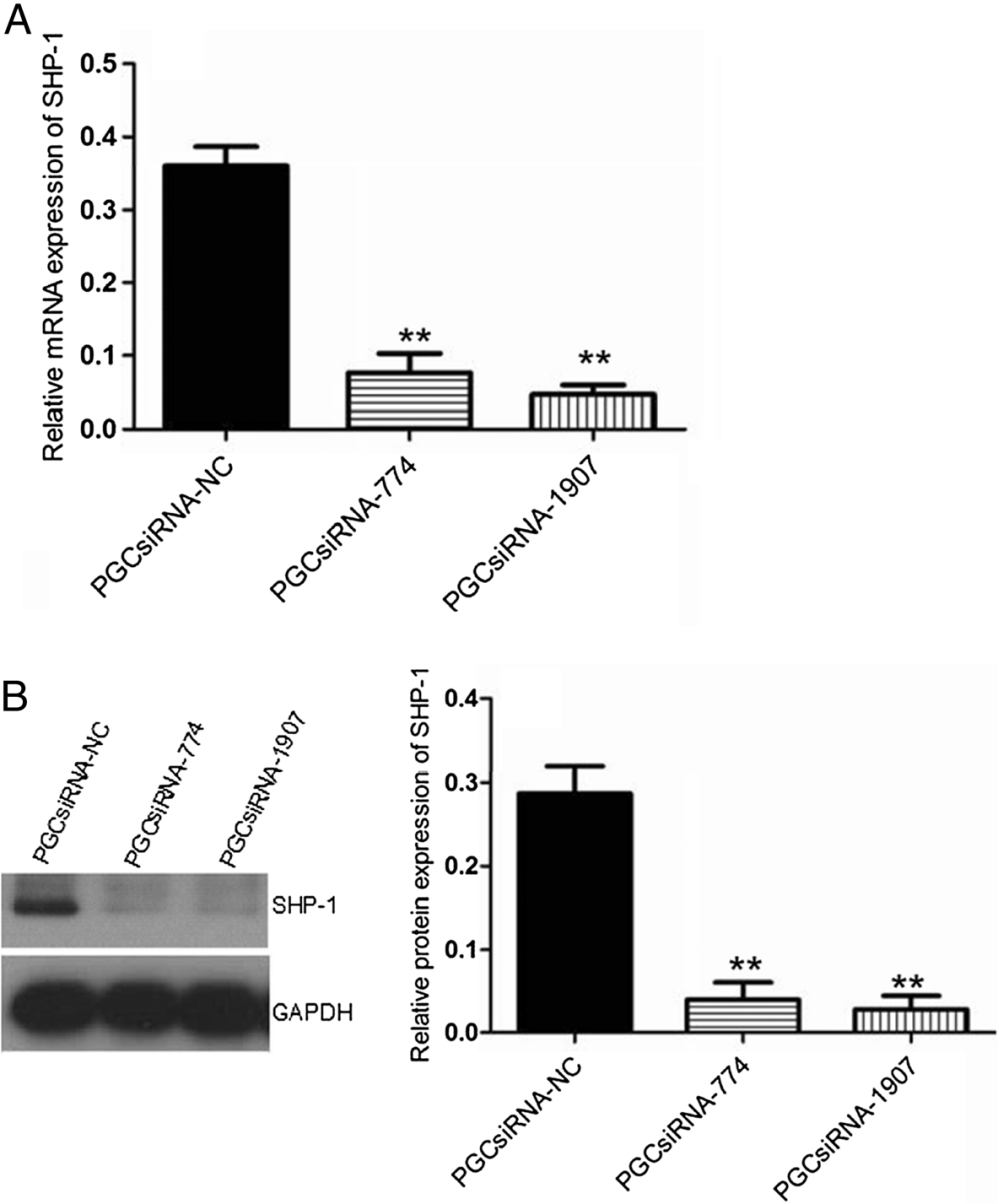
**Assessment of SHP1-siRNA efficiency. (A)** SHP1 mRNA expression in A549S1 cells was investigated by real-time RT-PCR following transfection with pGCsiRNA774 and pGCsiRNA1907 plasmids. **(B)** SHP1 protein expression in A549S1 cells was investigated by western blot following transfection with pGCsiRNA774 and pGCsiRNA1907 plasmids. GAPDH was used as an internal control (**P < 0.01 vs. the pGCsiRNA-NC group).

As shown in Figure 5, both pGCsiRNA774 and pGCsiRNA1907 plasmids containing SHP1 siRNA significantly inhibited SHP1 mRNA and protein expression by 89.3 ± 5.0% and 92.9 ± 2.2%, respectively, when compared with the control group (pGCsiRNA-NC).

### Effects of SHP1 siRNA on the expression of cell cycle-related proteins

**Figure 6:**
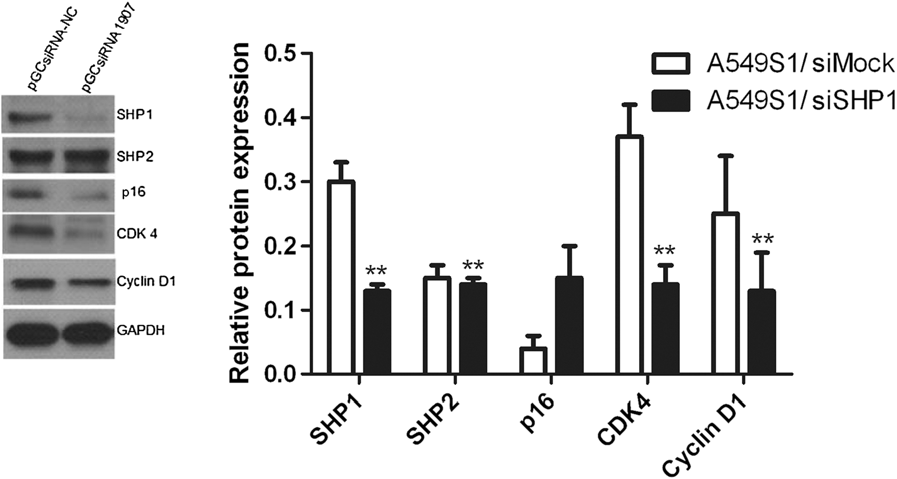
**Effects of stable SHP1 siRNA on the expression of cell cycle-related proteins.** Protein levels of SHP1, p16, CDK4 and Cyclin D1 were examined in A549S1/siSHP1 and A549S1/siMock by western blot. GAPDH was used as an internal control (*P < 0.05, **P < 0.01 vs. the A549S1/siMOCK group).

Positive clones for the pGCsiRNA1907 plasmid were cultured for three months using a compressing model in order to obtain a stably transfected A549S1 cell line (A549S1/siSHP1). Cells transfected with empty vector were regarded as A549S1/siMock. As shown in Figure 6, expression of SHP1, CDK4 and CylinD1 were down-regulated by 56.7%, 62.1% and 47.1%, respectively. Furthermore, p16 levels were increased by 3.39 folds in A549S1/siSHP1 cells compared with A549S1/siMock cells.

### Stable SHP1 siRNA expression increased the radiosensitivity of A549S1 cells

**Figure 7:**
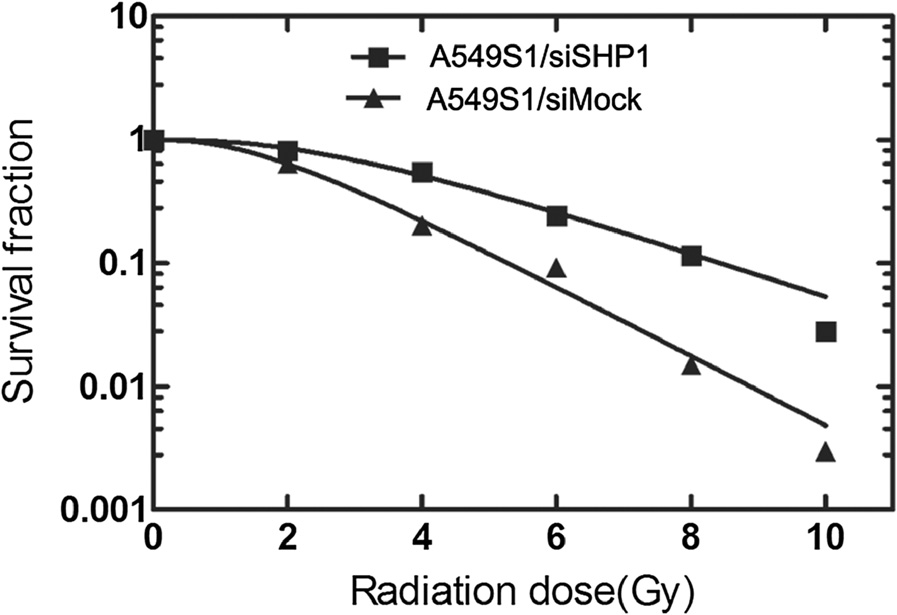
**Survival curves of A549S1/siSHP1 and A549S1/siMock cells.** Survival curves were obtained using the single-hit multi-target model (S = l-(1-e-D0/D)N) and the value of radiosensitivity parameters SF2, D0, Dq and N were calculated.

A549S1/siSHP1 cells were cultured for three months, and their survival fraction was determined by the clone formation assay along with cell survival curves, as shown in Figure 7. SF2, D0, Dq and N radiosensitivity parameters are listed in Table 4. Results showed that the radioresistance of A549S1/siSHP1 cells was significantly decreased compared with A549S1/siMock cells.

**Table 4:** Radiosensitivity parameters of A549S1/siSHP1 and A549S1/siMock cells

**Radiosensitivity parameters**	**A549S1/siMock**	**A549S1/siSHP1**
SF2	0.82	0.64
D0	1.99	1.75
Dq	1.97	1.56
N	4.28	2.80

SF2: cell survival fraction in 2 Gy irradiation dose; D0: mean lethal dose value; Dq: quasithreshold dose; N: extrapolation number (N).

### Effect of stable SHP1 siRNA inhibition on cell cycle

**Figure 8:**
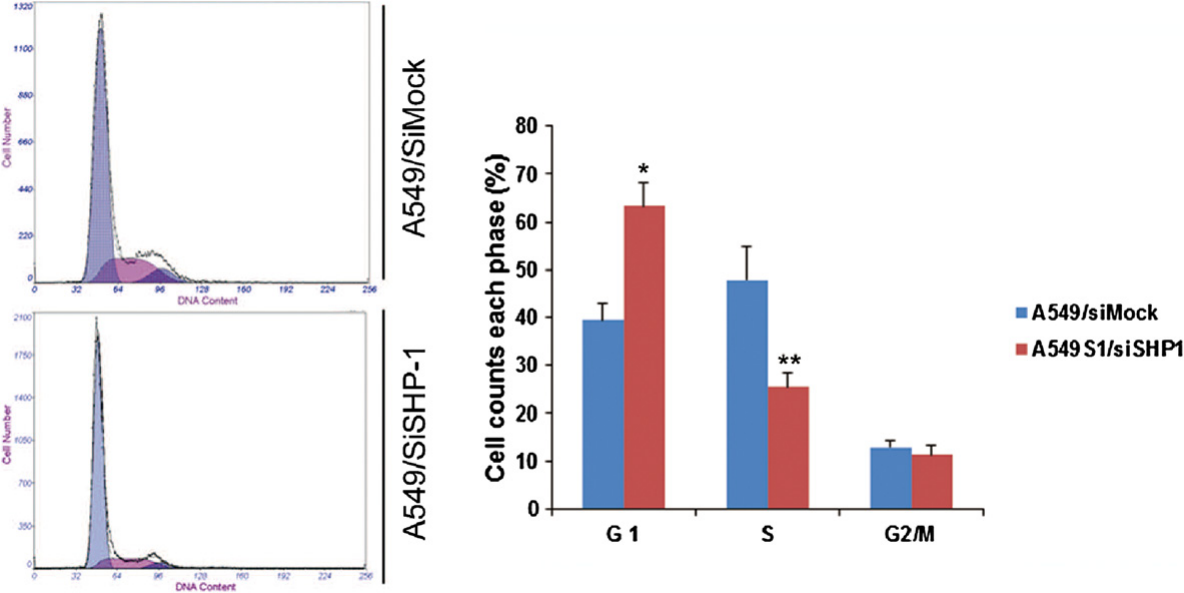
**Effects of stable SHP1 siRNA on cell cycle.** Cell cycle progression of A549S1/siSHP1 and A549/siMock were examined by FACS analysis (*P < 0.05, **P < 0.01 vs. A549S1/siMock).

As shown in Figure 8, the proportions of A549S1/siMock cells in G0/G1, S and G2/M phase were 39.4 ± 3.5%, 47.9 ± 7.1% and 12.7 ± 1.5%, respectively. In A549S1/siSHP1 cells, these proportions were changed to 63.3 ± 1.8%, 25.5 ± 2.8% and 11.3 ± 1.9%, respectively. These results showed that the proportion of cells in G0/G1 phase was significantly increased, while the proportion of cells in S phase was significantly decreased in A549S1/siSHP1 cells compared with A549S1/siMock cells (P < 0.01). However, there was no significant difference in the proportion of cells in G2/M phase between A549S1/siSHP1 and A549S1/siMock (P > 0.05).

## Discussion

It has been reported that a radioresistant phenotype in cells can be changed to a radiosensitive one during fractionated radiotherapy regimens, which could be beneficial for increasing treatment efficiency in destroying tumor cells. However, if the cell cycle does not change accordingly and effectively, this could lead to radioresistance. Ionizing radiations can induce changes in cell cycle progression, such as G1 and G2 phases arrest. Moreover, the arrest time is dependent on the intrinsic cell’s radiosensitivity, which is the key event for the cells to check the authenticity and integrity of their genetic material, and to repair damages. However, the detailed molecular mechanisms for this process are still unknown [25]. In this study, we investigated the relationship between radiosensitivity and cell cycle in NSCLC, as well as its molecular mechanisms.

We successfully established a NSCLC radioresistant cell line (A549S1 and S549S2) using two fractionated irradiation methods. However, the best irradiation dose and fractionation regimen for the generation of radiation-induced resistance are still controversial. Previous studies demonstrated that radioresistant subtype cell lines were rapidly established by 3–5 fractions of 6 cGy [26]. Thus, in this study, the A549S1 cell line was established by setting the irradiation dose at 6 cGy/fraction for 5 fractions. On the other hand, the control cell line A549S2 was established by the general fractionated irradiation method (2 cGy/fraction for 15 fractions). Results of this present study showed that the radiosensitivity of A549S1 was significantly decreased compared with A549 cells.

Previous studies have shown that there might be two reasons for the formation of radioresistant cells, including radiation-induced cell screening and cell mutation [26,27]. Indeed, radiosensitive cells are easily destroyed by radiations, while radioresistant cells survive in the harsh irradiated environment. In addition, radiations stimulate structural changes at the cellular and molecular levels, some of which induce a radioresistant phenotype by inducing mutations. Cells radiosensitivity is different with different cell cycle phases. For example, cells in S, G0/G1 and G2/M phases are radioresistant, relatively radiosenstive and radiosenstive, respectively [28].

Results from this study showed that the proportions of cells in the S and G0/G1 phases were significantly increased and decreased, respectively, in A549S1 cells compared with A549 cells. The proportions of cells in G2/M phase were not changed in these two cell lines, suggesting an S phase arrest in A549S1 cells. Moreover, the proportion of cells in each phase did not change in A549S2 cells. Thus, we hypothesized that the radioresistance of A549S1 was caused by changes in cell cycle progression. Our data confirmed that high-dose hypofractionated irradiation induced the formation of radioresistance in cells in vitro, which was related to the cell cyle in the tumor cells. These results are in line with previous findings [25,28]. In the meantime, protein expression levels of SHP1, CDK4 and CylinD1 were increased, while p16 expression level was decrased in A549S1 cells, suggesting an important role of these factors in radiosensitivity and cell cycle progression. Due to the fact that SHP2 expression was not changed in A549S1 cells compared with A549 cells, we investigated the effect of SHP1 siRNA on the regulation of radiosensitivity, as well as on p16, Cyclin D1 and CDK4 expression.

SHP-1 is closely related to the regulation of cell cycle. Indeed, a number of cellular proteins (such as protein kinase C, SHP1, Rac, Rho and Scr) are activators of the PI3K/Akt pathway, which has been demonstrated to play a crucial role in cell cycle progression [17,18]. Activation of the PI3K/Akt pathway increases Cyclin D1 and CDK4, and decreases p16 [17,18]. Cyclin D1 is an important regulator in the transformation of G1 to S phase. Normally, Cyclin D1 can bind with CDK4 to form a complex promoting Rb phosphorylation and stimulating cells transition from G1 into S phase. p16 is a tumor suppressor gene, which causes G0/G1 phase cell arrest by inhibiting Rb phosphorylation via p16 and Cyclin D1 competitory binding to CDK4. p16, Cyclin D1 and CDK4 are cell cycle regulatory factors, and their gene mutations or protein abnormalities are closely related to tumorigenesis, tumor development and progression in a variety of tumors [29]. However, even if these proteins play a clear role in tumorigenesis, the exact relationship between SHP1 and cell cycle-related proteins (Cyclin D1, CDK4 and p16) and its function in NSCLC or A549S1 cells is still unknown. Nevertheless, our results show that SHP1 kncokdown using siRNA increases p16 expression and decreases CDK4 and Cyclin D1 expressions, which may be mediated by the PI3K/Akt pathway, but how depletion of SHP1 results in an increase in p16 levels need to be further studied.

Our results showed that a stable SHP1 siRNA can induce a higher radiosensitivity in A549S1 cells. Compared with A549S1/siMock cells, the proportions of cells in S and G0/G1 phases were significantly decreased and increased in A549S1/siSHP1cells, respectively. Meanwhile, the inhibition of SHP1 induced p16 up-regulation, and CDK4 and CylinD1 down-regulation. These data suggested that stable inhibition of SHP1 increased the radiosensitivity by affecting the expression of CDK4, CylinD1 and p16 (cell cycle related proteins), thus delaying the G1/S checkpoint in NSCLC cells. Our results are supported by the recent observations indicating that SHP1 supression induces G1/S arrest in prostate cancer cells [18].

## Conclusions

In summary, we observed that SHP-1 attenuated the radiosensitivity of NSCLC cells through affecting cell cycle induced by cell cycle-related proteins such as CDK4, CylinD1 and p16. These findings could potentially increase our understanding of the molecular mechanisms involved in this process, and could identify potential targets to promote the efficacy of radiotherapy in NSCLC.

## Competing interests

The authors declare that they have no conflict of interest.

## Authors’ contributions

RC and QD carried out the studies, participated in collecting data, and drafted the manuscript. GP performed the statistical analysis and participated in its design. PL, JX, ZZ, JH helped to draft the manuscript. All authors read and approved the final manuscript.
